# Oxygen desaturation during the six-minute walk test in COPD patients*

**DOI:** 10.1590/S1806-37132014000300004

**Published:** 2014

**Authors:** Maria Ângela Fontoura Moreira, Gabriel Arriola de Medeiros, Francesco Pinto Boeno, Paulo Roberto Stefani Sanches, Danton Pereira da Silva, André Frotta Müller

**Affiliations:** Pulmonary Physiology Clinic, Department of Pulmonology, Porto Alegre Hospital de Clínicas, Porto Alegre, Brazil; Pulmonary Physiology Clinic, Department of Pulmonology, Porto Alegre Hospital de Clínicas, Porto Alegre, Brazil; Pulmonary Physiology Clinic, Department of Pulmonology, Porto Alegre Hospital de Clínicas, Porto Alegre, Brazil; Department of Research and Development in Biomedical Engineering, Porto Alegre Hospital de Clínicas, Porto Alegre, Brazil; Department of Research and Development in Biomedical Engineering, Porto Alegre Hospital de Clínicas, Porto Alegre, Brazil; Department of Research and Development in Biomedical Engineering, Porto Alegre Hospital de Clínicas, Porto Alegre, Brazil

**Keywords:** Pulmonary disease, chronic obstructive, Exercise test, Blood gas monitoring, transcutaneous

## Abstract

**Objective:**

To evaluate the behavior of oxygen saturation curves throughout the six-minute walk test (6MWT) in patients with COPD.

**Methods:**

We included 85 patients, all of whom underwent spirometry and were classified as having moderate COPD (modCOPD, n = 30) or severe COPD (sevCOPD, n = 55). All of the patients performed a 6MWT, in a 27-m corridor with continuous SpO_2_ and HR monitoring by telemetry. We studied the SpO_2_ curves in order to determine the time to a 4% decrease in SpO_2_, the time to the minimum SpO_2_ (Tmin), and the post-6MWT time to return to the initial SpO_2_, the last designated recovery time (RT). For each of those curves, we calculated the slope.

**Results:**

The mean age in the modCOPD and sevCOPD groups was 66 ± 10 years and 62 ± 11 years, respectively. At baseline, SpO_2_ was > 94% in all of the patients; none received supplemental oxygen during the 6MWT; and none of the tests were interrupted. The six-minute walk distance did not differ significantly between the groups. The SpO_2_ values were lowest in the sevCOPD group. There was no difference between the groups regarding RT. In 71% and 63% of the sevCOPD and modCOPD group patients, respectively, a ≥ 4% decrease in SpO_2_ occurred within the first minute. We found that FEV_1_% correlated significantly with the ΔSpO_2_ (r = −0.398; p < 0.001), Tmin (r = −0.449; p < 0.001), and minimum SpO_2_ (r = 0.356; p < 0.005).

**Conclusions:**

In the sevCOPD group, in comparison with the modCOPD group, SpO_2_ was lower and the Tmin was greater, suggesting a worse prognosis in the former.

## Introduction

Advances in research on and in the treatment and diagnosis of lung diseases have shown the importance of including the six-minute walk test (6MWT) in the functional assessment of lung disease patients, more specifically in the detection of exercise-induced hypoxemia, which is considered an important marker of respiratory disease severity. The acquisition of reproducible measurements is necessary for this assessment.^(^ ^1^ ^-^ ^5^ ^)^

The 6MWT is widely requested since, in addition to being easy to administer, inexpensive, and well-tolerated by the patient, it is the mode of submaximal exercise that most closely approximates activities of daily living. It is attractive because it combines ease of performance and operational simplicity. Therefore, it is usually used as an adjunctive tool in the assessment of COPD, cystic fibrosis, heart disease, peripheral vascular disease, etc.^(^ ^2^ ^-^ ^4^ ^,^ ^6^ ^)^

The American Thoracic Society guidelines recommend that the 6MWT be performed indoors, along a flat, straight, 30-m track, on which the patient should walk for six minutes, with the aim of covering the greatest distance possible.^(^ ^7^ ^)^

The 6MWT is among the most commonly used tests to assess exercise tolerance in individuals with chronic obstructive disease and individuals with interstitial disease. Such patients may experience a significant decrease in SpO_2_ during submaximal exercise or even desaturation at rest. Exertional hypoxemia can be explained by pathophysiological factors, such as airflow limitation, imbalance between oxygen supply and consumption, systemic inflammation, and oxidative stress, affecting peripheral muscle oxygenation. The significant decrease in the levels of circulating oxygen, resulting from the increased demand caused by the effort put forth, can lead to increased blood pressure, increased dyspnea, and increased muscle fatigue, thereby reducing submaximal exercise tolerance.^(^ ^8^ ^)^

Patients with COPD do not show the same limitation during exercise or activities of daily living. Exercise performance and exercise maintenance depend primarily on flawless interaction among the systems that control ventilation, gas exchange, blood flow, hemoglobin, oxygen/carbon dioxide transport, oxygen use, and carbon dioxide production.^(^ ^8^ ^)^

In patients with COPD, one of the most important adverse events during the 6MWT is oxygen desaturation, which can be more accurately assessed if there is continuous monitoring throughout the test. Therefore, the objective of the present study was to evaluate the behavior of oxygen saturation curves throughout the 6MWT in patients with COPD.

## Methods

The data were collected between January and December of 2012 in the Pulmonary Physiology Clinic of the Department of Pulmonology of the *Hospital de Clínicas de Porto Alegre* (HCPA, Porto Alegre *Hospital de Clínicas*), located in the city of Porto Alegre, Brazil. This study was analyzed and approved by the HCPA Health Research Ethics Committee (Project no. 09-549), and the patients invited to participate in the study gave written informed consent before performing the 6MWT.

We included male and female patients who had been diagnosed with COPD,^(^ ^9^ ^)^ were stable, and had spirometry results indicative of moderate COPD (modCOPD) or severe COPD (sevCOPD), as classified by the 2002 Brazilian Thoracic Association Guidelines for Pulmonary Function Tests.^(^ ^10^ ^)^ Spirometry was performed by spirometry technicians certified by the Brazilian Thoracic Association. A spirometer (Jaeger, Würtzburg, Germany) was used, and the predicted values of Crapo were employed.^(^ ^11^ ^)^ Spirometry was performed 1 h before the 6MWT on the same day. Values of FEV_1_ and VC were obtained from the flow-volume curves.

**Figure 1:**
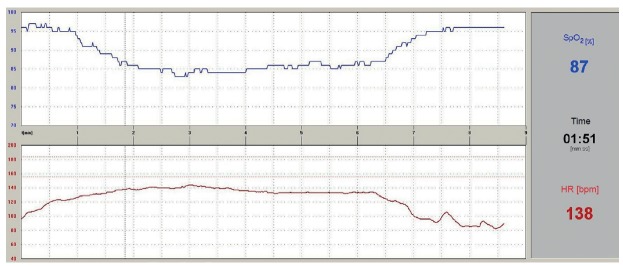
Monitoring of HR and SpO_2_.

The 6MWT was conducted in a 27-m corridor in accordance with the America Thoracic Society guidelines.^(^ ^7^ ^)^ At the HCPA, it is possible to monitor HR and SpO_2_ continuously by telemetry throughout the 6MWD with the use of a digital oximetry module and of a software program developed by the Biomedical Engineering team at HCPA. This system allows the simultaneous transfer of HR and SpO_2_ data to the computer, making it possible to monitor the degree of oxygen desaturation in real time, which thereby allows a better assessment of the degree of disease severity.^(^ ^5^ ^,^ ^7^ ^)^ Figure 1 shows a recorded curve. All of the included patients completed the 6MWT without interruption and had a baseline SpO_2_ > 94%. None of the patients received supplemental oxygen during the test. We excluded from the sample those with orthopedic impairments, interstitial diseases, or pulmonary arterial hypertension, or with any condition that would compromise their ability to perform the 6MWT. The curves showing a ≥ 4% decrease in SpO_2_ were analyzed.

We studied the SpO_2_ curves in order to determine the time to a 4% decrease in SpO_2_ and the time to the minimum SpO_2_, as well as the post-6MWT time to return to the initial SpO_2_, designated recovery time. We calculated the slope of each of those curves with the following formula: (final SpO_2_ - initial SpO_2_) ÷ Δtime between those points

**Figure 2:**
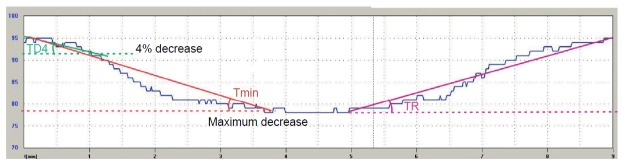
Example of slopes of the curves. TD4: time to desaturation of 4%. Tmin: time to the minimum SpO_2_; and RT: recovery time (i.e., time to return to the initial SpO_2_).

The slopes were compared to determine changes in them because of the severity of airway obstruction. Figure 2 shows an example of the slopes calculated.

The statistical analysis of the collected data was performed with the Statistical Package for the Social Sciences, version 18.0 (SPSS Inc., Chicago, IL, USA). Data were analyzed for normality and homogeneity of variance. The independent sample t-test was used for the comparison between the two groups. Pearson's correlation test was used for analysis of correlations. For all analyses, the level of significance was set at p < 0.05. Values are expressed as means and standard deviations.

## Results

The study sample consisted of 85 patients: 55 with sevCOPD (mean age of 62 ± 11.3 years and mean body mass index [BMI] of 22.5 ± 3.3 kg/m^2)^; and 30 with modCOPD (mean age of 66.0 ± 10.1 years and BMI of 25.1 ± 2.8 kg/m^2)^. Table 1 shows the variables assessed in the two groups.

**Table 1:** General characteristics of the study population

Variable	modCOPD	sevCOPD	p
	(n = 30)	(n = 55)	
BMI, kg/m^2^	25.1 ± 2.8	22.5 ± 3.3	0.01*
Age, years	66 ± 10	62 ± 11	0.11
Pre-6MWT SpO_2_, %	95.0 ± 1.9	95.0 ± 2.1	0.85
Minimum SpO_2_, %	87.3 ± 3.4	85.0 ± 4.3	0.01*
TD4, s	64 ± 36	66 ± 41	0.81
Tmin, s	109 ± 55	168 ± 67	0.01*
RT, s	112.6 ± 28.8	120.2 ± 31.5	0.28
FEV1%	53.1 ± 9.6	28.0 ± 6.2	0.01*
6MWD, m	451 ± 73	435 ± 74	0.35
Slope of the line for a ≥ 4% decrease in SpO_2_	0.08 ± 0.04	0.08 ± 0.04	0.89
Slope of the line for maximum decrease in SpO_2_	0.08 ± 0.03	0.07 ± 0.05	0.27
Slope of the line for return to the initial SpO_2_	0.07 ± 0.02	0.09 ± 0.04	0.05

**modCOPD:** : moderate COPD

**sevCOPD:** : severe COPD

BMI : body mass index

6MWT : six-minute walk test

TD4 : time to desaturation of ≥ 4%

Tmin : time to the minimum SpO_2_

RT : recovery time (i.e., time to return to the initial SpO_2_)

6MWD : six-minute walk distance

The groups were found to be homogeneous with respect to age and pre-6MWT SpO_2_. In neither of the groups did the BMI exceed 30 kg/m², a value above which spirometry results are affected.^(^ ^10^ ^)^ The six-minute walk distance did not differ significantly between the groups. The minimum SpO_2_ was significantly lower in the sevCOPD group (p < 0.014). A 4% decrease in SpO_2_ occurred within the first minute in 63% and 71% of the modCOPD and sevCOPD group patients, respectively. The time to desaturation of 4% and the recovery time did not differ significantly between the groups; however, the time to the minimum SpO_2_ was greater in the sevCOPD group than in the modCOPD group (p < 0.001). The slopes of the SpO_2_ curves for desaturation of 4%, maximum decrease, and recovery were not found to differ significantly between the groups. The change in SpO_2_ (ΔSpO_2_) between the baseline value and the maximum decrease was statistically different between the two groups (p = 0.005).

We found that FEV_1_% showed a moderate positive correlation with the minimum SpO_2_ (r = 0.356; p < 0.005), a moderate negative correlation with the ΔSpO_2_ (r = −0.398; p < 0.001), and a moderate negative correlation with the time to the minimum SpO_2_ (r = −0.449; p < 0.001).

**Figure 3:**
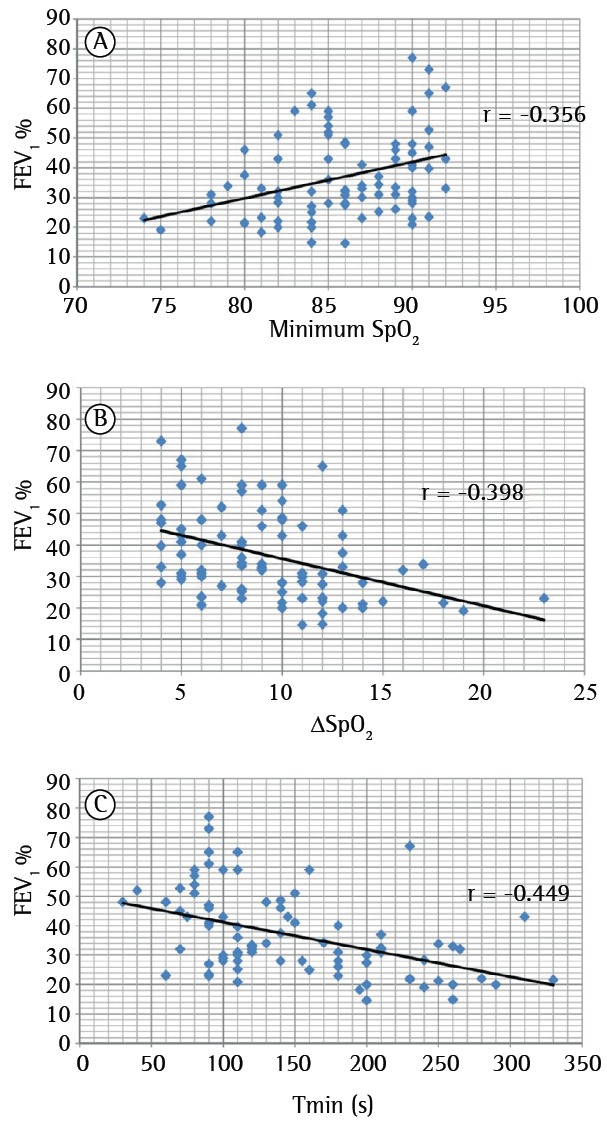
Correlations of changes in FEV_1_% with the minimum SpO_2_ (in A), ΔSpO_2_ (in B), and time to the minimum SpO_2_ (T_min_; in C).

The slope of the maximum decrease in SpO_2_ showed a moderate negative correlation with the time to the minimum SpO_2_ (r = −0,467; p < 0,001), time to desaturation of 4% (r = −0.437; p < 0.001), and minimum SpO_2_ (r = −0.393; p < 0.001). The six-minute walk distance (6MWD) showed no significant correlations with SpO_2_ or its variations or with FEV_1_%. 

## Discussion

Exercise-induced desaturation can be measured in the 6MWT and is an index that has prognostic value in interstitial diseases and COPD. A ≥ 4% decrease in SpO_2_ suggests significant desaturation and is used for assessing the need for oxygen supplementation in patients with chronic lung disease.^(^ ^12^ ^)^ Another index of functional capacity is the 6MWD, which has prognostic value in COPD.^(^ ^13^ ^)^

However, hypoxemia is a major problem in respiratory medicine, since it is very common in patients with lung disease and must be rapidly assessed and treated to prevent irreversible organ damage.

Exercise-induced desaturation is commonly observed in patients with COPD; however, clinical parameters cannot identify such change. A resting SpO_2_ of < 95% has been reported to be a predictor of exercise-induced desaturation, especially in patients with a ≥ 36% reduction in DLCO^(^ ^14^ ^)^ Zafar et al.^(^ ^15^ ^)^ found no significant correlation between changes (decreases) in SpO_2_ and resting SpO_2_. Our study also found no significant correlation between baseline SpO_2_ and decreases in SpO_2_ (r = 0.08; p = 0.46).

The basis on which the theory of exercise intolerance in COPD is built is multifactorial: increased respiratory muscle work and oxygen uptake; lower limb skeletal muscle dysfunction; and dynamic lung hyperinflation; acting either alone or in combination.^(^ ^8^ ^)^ Zafar et al.,^(^ ^15^ ^)^ studying 30 patients with COPD, reported a good correlation between oxygen desaturation during the 6MWT and dynamic hyperinflation, but no correlation with the 6MWD. Our results also showed no correlation between desaturation and the 6MWD; however, we did not assess hyperinflation in the present study.

We found that the 6MWD showed no correlation with changes in SpO_2_. There have been reports of skeletal muscle changes in patients with COPD, with the predominance of glycolytic fibers over oxidative fibers being highlighted. As a result, patients predominantly use the anaerobic metabolism at a low level of exercise,^(^ ^16^ ^,^ ^17^ ^)^ characterizing a change in the metabolic pathway and reducing the aerobic load. The occurrence of some factors, such as inflammatory stress, physical deconditioning, prolonged use of corticosteroids, and hypoxemia, contributes to altering muscle contractile activity, triggering a series of adaptations that involve muscle fiber changes. According to one group of authors,^(^ ^18^ ^)^ the work of breathing in the group of COPD patients who recruit abdominal muscles is twice that in the group of COPD patients who do not do so, being associated with increased dyspnea and decreased exercise tolerance. This is a possible explanation for our results, since patients may have a predominance of glycolytic fibers, may not recruit abdominal muscles, or both.

Previous studies^(^ ^19^ ^,^ ^20^ ^)^ have shown that the time to desaturation during the 6MWT is an indicator of the possibility of desaturation during activities of daily living, culminating in severe hypoxemia and the need for oxygen therapy. Jenkins & Cecins^(^ ^21^ ^)^ analyzed the adverse events that occurred during the 6MWT in a group of 572 patients with COPD who completed the 6MWT; 345 (47%) of the patients experienced significant desaturation (a ≥ 4% decrease). The study by Jenkins & Cecins^(^ ^21^ ^)^ highlights the importance of continuous monitoring SpO_2_ during the 6MWT. The telemetry system used at the HCPA enabled us to monitor the behavior of SpO_2_ during the 6MWT in real time.

One group of authors^(^ ^20^ ^)^ showed that, of 83 patients with COPD who performed the 6MWT, 48 experienced early desaturation (SpO_2_ < 90% before the first minute) and that, over a 5-year follow-up period, 65% of those patients developed severe hypoxemia and required home oxygen therapy, compared with 11% of the patients who did not experience early desaturation (p < 0.001). Early desaturation is also associated with desaturation during a 24-h period and during most activities of daily living. In our sample of patients who experienced desaturation during the 6MWT, we noticed that most experienced desaturation of ≥ 4% within the first minute (71% and 63% of the sevCOPD and modCOPD group patients, respectively), which indicates the need for a more rigorous assessment of the routine activities of these individuals.

In one study,^(^ ^22^ ^)^ 224 patients with COPD were divided into two groups: those with and those without oxygen desaturation during the 6MWT. The patients were followed for 3 years, and the desaturation group was found to have a more rapid decline in FEV_1_ (p = 0.006), which suggests that exercise-induced desaturation can be a predictor of pulmonary function decline in patients with COPD. In our study, FEV_1_ was found to be a good indicator of exercise-induced desaturation, showing a significant moderate negative correlation with the ΔSpO_2_ (r = −0.398; p < 0.001) and time to the minimum SpO_2_ (r = −0.448; p < 0.001).

Oxygen desaturation is a monitoring parameter that qualifies the performance of patients on the 6MWT and aids in determining the degree of disease-related impairment during physical exertion. Analysis of desaturation curves allows a comprehensive view of the time to a decrease in SpO_2_, the intensity of that decrease, and the recovery time, which can assist in determining clinical severity. However, to our knowledge, no other studies have reported this type of data, which precludes a comparison with our results.

The present study underscores the importance of oxygen desaturation analysis with continuous monitoring during the 6MWT in patients with COPD. In the sevCOPD group, in comparison with the modCOPD group, SpO_2_ was lower and most patients experienced early desaturation (within the first minute), suggesting a worse prognosis. The FEV_1_ variable was found to be a good marker of exercise-induced desaturation, showing a moderate correlation with the minimum SpO_2_, ΔSpO_2_, and time to the minimum SpO_2_.

## References

[1] Brunetto AF, Pitta FO, Probst VS, Paulin E, Yamaguti WP, Ferreira LF (2003). Influência da saturação de O_2_ na velocidade
do teste de distância percorrida de seis minutos em pacientes com DPOC
grave. Rev Bras Fisioter.

[2] Rondelli RR, Oliveira AN, Dal Corso S, Malaguti C (2009). Uma atualização e proposta de padronização do teste de
caminhada de seis minutos. Fisioter Movimento.

[3] Pires SR, Oliveira AC, Perreira VF, Britto RR (2007). Teste de caminhada de seis minutos em diferentes faixas
etárias e índice de massa corporal. Rev Bras Fisioter.

[4] Ziegler B, Rovedder PM, Lukrafka JL, Oliveira CL, Menna-Barreto SS, Dalcin Pde T (2007). Submaximal exercise capacity in adolescent and adult
patients with cystic fibrosis. J Bras Pneumol.

[5] Dumke A (2006). Estudo do comportamento da saturação periférica de oxigênio
durante o teste de caminhada de 6 minutos em pacientes com doenças pulmonares
crônicas.

[6] Rodrigues SL, Mendes HF, Viegas CA (2004). Teste da caminhada de seis minutos: estudo do efeito do
aprendizado em portadores de doença pulmonar obstrutiva crônica. J Pneumol.

[7] ATS Committee on Proficiency Standards for Clinical Pulmonary Function
Laboratories (2002). ATS statement: guidelines for the six-minute walk
test. Am J Respir Crit Care Med.

[8] Russo R, Iamonti VC, Jardim JR (2012). Intolerância ao exercício no paciente com
DPOC. Pneumol Paulista.

[9] Global Initiative for Chronic Obstructive Lung Disease (2013). Global Strategy for the Diagnosis, Management and Prevention of
Chronic Obstructive Pulmonary Disease.

[10] Sociedade Brasileira de Pneumologia e Tisiologia (2002). Diretrizes para testes de função
pulmonar. J Pneumol.

[11] Crapo RO, Morris AH, Gardner RM (1981). Reference spirometric values using techniques and
equipment that meet ATS recommendations. Am Rev Respir Dis.

[12] Puente Maestú L, García de Pedro J (2012). Lung function tests in clinical
decision-making. Arch Bronconeumol.

[13] Hagarty EM, Skorodin MS, Langbein WE, Hultman CI, Jessen JA, Maki KC (1997). Comparison of three oxygen delivery systems during
exercise in hypoxemic patients with chronic obstructive pulmonary
disease. Am J Respir Crit Care Med.

[14] Knower MT, Dunagan DP, Adair NE, Jr Chin R (2001). Baseline oxygen saturation predicts exercise
desaturation below prescription threshold in patients with chronic obstructive
pulmonary disease. Arch Intern Med.

[15] Zafar MA, Tsuang W, Lach L, Eschenbacher W, Panos RJ (2013). Dynamic Hyperinflation correlates with exertional oxygen
desaturation in patients with chronic obstructive pulmonary
disease. Lung.

[16] Gosker HR, van Mameren H, van Dijk PJ, Engelen MP, van der Vusse GJ, Wouters EF (2002). Skeletal muscle fibre-type shifting and metabolic
profile in patients with chronic obstructive pulmonary disease. Eur Respir J.

[17] Engelen MP, Schols AM, Does JD, Gosker HR, Deutz NE, Wouters EF (2000). Exercise-induced lactate increase in relation to muscle
substrates in patients with chronic obstructive pulmonary disease. Am J Respir Crit Care Med.

[18] Aliverty A, Macklem PT (1985). Last Word on Point:Counterpoint: The major limitation to
exercise performance in COPD is 1) inadequate energy supply to the respiratory and
locomotor muscles, 2) lower limb muscle dysfunction, 3) dynamic
hyperinflation. J Appl Physiol.

[19] García-Talavera I, García CH, Macario CC, de Torres JP, Celli BR, Aguirre-Jaime A (2008). Time to desaturation in the 6-min walking distance test
predicts 24-hour oximetry in COPD patients with a PO_2_ between 60 and
70mmHg. Respir Med.

[20] Garcia-Talavera I, Tauroni A, Trujillo JL, Pitti R, Eiroa L, Aguirre-Jaime A (2011). Time to desaturation less than one minute predicts the
need for long-term home oxygen therapy. Respir Care.

[21] Jenkins S, Cecins N (2011). Six-minute walk test: observed adverse events and oxygen
desaturation in a large cohort of patients with chronic lung
disease. Intern Med J.

[22] Kim C, Seo JB, Lee SM, Lee JS, Huh JW, Lee JH (2013). Exertional desaturation as a predictor of rapid lung
function decline in COPD. Respiration.

