# Supplementary material for: Oxygen desaturation during the six-minute walk test in COPD patients
Source: J Bras Pneumol. 2014 May-Jun;40(3):222–8. doi: 10.1590/S1806-37132014000300004 (PMC4109193; doi:10.1590/S1806-37132014000300004)
Supplement: Translation

# Translation: Análise da dessaturação de oxigênio durante o teste de caminhada de seis minutos em pacientes com DPOC*

**Authors:** Maria Ângela Fontoura Moreira, Gabriel Arriola de Medeiros, Francesco Pinto Boeno, Paulo Roberto Stefani Sanches, Danton Pereira da Silva, André Frotta Müller

**Affiliations:** Unidade de Fisiologia Pulmonar, Serviço de Pneumologia, Hospital de Clínicas de Porto Alegre, Porto Alegre (RS) Brasil; Unidade de Fisiologia Pulmonar, Serviço de Pneumologia, Hospital de Clínicas de Porto Alegre, Porto Alegre (RS) Brasil; Unidade de Fisiologia Pulmonar, Serviço de Pneumologia, Hospital de Clínicas de Porto Alegre, Porto Alegre (RS) Brasil; Serviço de Pesquisa e Desenvolvimento em Engenharia Biomédica, Hospital de Clínicas de Porto Alegre, Porto Alegre (RS) Brasil; Serviço de Pesquisa e Desenvolvimento em Engenharia Biomédica, Hospital de Clínicas de Porto Alegre, Porto Alegre (RS) Brasil; Serviço de Pesquisa e Desenvolvimento em Engenharia Biomédica, Hospital de Clínicas de Porto Alegre, Porto Alegre (RS) Brasil

**Keywords:** Doença pulmonar obstrutiva crônica, Teste de esforço, Monitorização transcutânea dos gases sanguíneos

## Abstract

**Objetivo:**

Avaliar o comportamento da curva de saturação de oxigênio durante o teste de caminhada de seis minutos (TC6) em pacientes com DPOC.

**Métodos:**

Incluímos 85 pacientes e todos realizaram espirometria, sendo classificados como portadores de DPOC moderada (DPOCm, n = 30) ou grave (DPOCg, n = 55). Todos os pacientes realizaram TC6 em um corredor de 27 m com monitoramento contínuo da SpO_2_ e FC por telemetria. A partir das curvas de SpO_2_, foram analisados os tempos para atingir a queda de 4% da SpO_2_, para atingir a SpO_2_ mínima (Tmin) e para a recuperação da SpO_2_ após o TC6 (TR). Foram calculadas as inclinações dessas curvas.

**Resultados:**

A média de idade nos grupos DPOCm e DPOCg foi de 62 ± 11 anos e 66 ± 10 anos, respectivamente. Todos os pacientes iniciaram o teste com SpO_2_ > 94%, nenhum recebeu suplementação de oxigênio durante o TC6, e não houve interrupções. A distância percorrida no TC6 não apresentou diferença significativa entre os grupos. Os menores valores da SpO_2_ ocorreram no grupo DPOCg. Não houve diferença no TR entre os grupos, e 71% e 63% dos pacientes nos grupos DPOCg e DPOCm, respectivamente, apresentaram queda de SpO_2_ ≥ 4% até o primeiro minuto. O VEF_1_% apresentou correlações significativas com ΔSpO_2_ (r = −0,398; p < 0,001), Tmin (r = −0,449; p < 0,001) e SpO_2_ mínima (r = 0,356; p < 0,005).

**Conclusões:**

As curvas dos pacientes do grupo DPOCg em relação às do grupo DPOCm apresentaram valores menores de SpO_2_ e maior Tmin, sugerindo um pior prognóstico nos primeiros.

## Introdução

Avanços nas pesquisas, no tratamento e no diagnóstico de doenças pulmonares vêm demonstrando a importância da inclusão do teste da caminhada de seis minutos (TC6) na avaliação funcional de pacientes com pneumopatias, mais precisamente na detecção da hipoxemia induzida pelo exercício, que é considerada um importante marcador da gravidade das doenças respiratórias. A promoção de medidas reprodutíveis para essa avaliação torna-se necessária.^(^ ^1^ ^-^ ^5^ ^)^

O TC6 é amplamente solicitado, pois além de ser de fácil aplicação, ter baixo custo e ser bem tolerado pelo paciente, é o exercício de capacidade submáxima que melhor representa atividades da vida diária. Dessa maneira, torna-se atrativo por conciliar facilidade de execução e simplicidade operacional. Assim, é normalmente aplicado como complemento na avaliação de DPOC, fibrose cística, cardiopatias, doenças vasculares periféricas, entre outras.^(^ ^2^ ^-^ ^4^ ^,^ ^6^ ^)^

As diretrizes da *American Thoracic Society,* com relação ao TC6, recomendam a utilização de um espaço físico fechado e plano com uma distância reta de 30 m, onde o paciente deverá caminhar por seis minutos, com o objetivo de percorrer a maior distância possível.^(^ ^7^ ^)^

O TC6 está entre os testes mais utilizados para avaliar a tolerância ao exercício em indivíduos portadores de doenças obstrutivas e crônicas e de doenças intersticiais. Esses pacientes podem apresentar um decréscimo acentuado da SpO_2_ quando submetidos a exercícios submáximos ou mesmo dessaturação em repouso. A hipoxemia decorrente do esforço pode ser explicada por fatores fisiopatológicos, como limitação ventilatória, desproporção entre a oferta e o consumo de oxigênio, inflamação sistêmica e estresse oxidativo, afetando a oxigenação muscular periférica. A queda significativa nos níveis de oxigênio circulante, resultante do aumento da demanda pelo esforço realizado, pode ocasionar o aumento da pressão arterial, da dispneia e da fadiga muscular, reduzindo assim a tolerância na execução de exercícios submáximos.^(^ ^8^ ^)^

Os pacientes com DPOC não apresentam a mesma limitação ao realizar exercícios ou atividades da vida diária. O desempenho e a manutenção do exercício dependem basicamente da interação perfeita entre os sistemas que comandam e controlam a ventilação, a troca gasosa, o fluxo de sangue, a hemoglobina, o transporte de oxigênio/dióxido de carbono, a utilização de oxigênio e a produção de dióxido de carbono.^(^ ^8^ ^)^

No TC6 dos pacientes com DPOC, um dos eventos adversos mais importantes é a dessaturação de oxigênio, que pode ser mais bem avaliada se houver um monitoramento continuo durante o teste. Logo, o objetivo do presente estudo foi analisar o comportamento da curva de saturação de oxigênio durante o TC6 em pacientes com DPOC.

## Métodos

Os dados foram coletados no período entre janeiro e dezembro de 2012 na Unidade de Fisiologia Pulmonar do Serviço de Pneumologia do Hospital de Clínicas de Porto Alegre (HCPA), na cidade de Porto Alegre (RS). A pesquisa foi analisada e aprovada pelo Comitê de Pesquisa e Ética em Saúde do HCPA (Projeto 09-549), e os pacientes convidados a participar da pesquisa assinaram o termo de consentimento livre e esclarecido antes da realização do TC6.

Foram incluídos no estudo pacientes com diagnóstico de DPOC,^(^ ^9^ ^)^ de ambos os sexos, estáveis, cuja espirometria indicava obstrução moderada (DPOCm) ou grave (DPOCg), classificados de acordo com as Diretrizes Brasileiras para Testes de Função Pulmonar de 2002.^(^ ^10^ ^)^ As espirometrias foram realizadas por técnicos aprovados como técnicos em espirometria pela Sociedade Brasileira de Pneumologia e Tisiologia. Utilizou-se um espirômetro (Jaeger, Würtzburg, Alemanha) e foram adotados os valores previstos de Crapo.^(^ ^11^ ^)^ A espirometria foi realizada 1 h antes do TC6 no mesmo dia. O VEF_1_ e a CV foram retirados das curvas fluxo-volume.

O TC6 foi realizado em um corredor de 27 m seguindo as normas da *American Thoracic Society*.^(^ ^7^ ^)^ No HCPA, é possível a monitorização contínua da FC e da SpO_2_ durante o TC6, através de telemetria, utilizando um módulo de oximetria digital e um *software* desenvolvido pela Engenharia Biomédica do HCPA. Esse sistema permite a transferência simultânea da FC e SpO_2_ para o computador, tornando possível a observação do grau de dessaturação de oxigênio em tempo real, permitindo assim uma melhor avaliação do nível de gravidade da doença.^(^ ^5^ ^,^ ^7^ ^)^ A Figura 1 mostra o registro de uma curva. Todos os pacientes incluídos completaram o TC6 sem interrupção e iniciaram o teste com valores de SpO_2_ > 94%. Nenhum paciente recebeu suplementação de oxigênio durante o esforço. Foram excluídos da amostra aqueles que apresentavam patologias ortopédicas, doenças intersticiais e hipertensão arterial pulmonar associada ou qualquer impeditivo que limitasse a execução do TC6. Foram analisadas as curvas nas quais houve uma queda da SpO_2_ ≥ 4%.

**Figura 1:** Monitoramento da FC e da SpO2.

A partir das curvas de SpO_2_ foi determinado o tempo para a queda de 4% na SpO_2_ e para atingir o menor nível de saturação, como também o tempo de recuperação após os 6 min. A inclinação dessas curvas foi calculada através da seguinte fórmula: 

(SpO_2_ final - SpO_2_ inicial) ÷ Δtempo entre esses momentos

As inclinações foram comparadas a fim de se determinar mudanças nessas em função da gravidade da obstrução. A Figura 2 mostra um exemplo das inclinações calculadas.

**Figura 2:** Exemplo de inclinação das curvas. TD4: tempo para atingir uma dessaturação de 4%. Tmin: tempo para atingir SpO2 mínima; e TR: tempo de recuperação da SpO2.

A análise estatística dos dados coletados foi realizada com o programa *Statistical Package for the Social Sciences*, versão 18.0 (SPSS Inc., Chicago, IL, EUA). A normalidade e a homogeneidade da variância dos dados foram analisadas. Foi utilizado o teste t para amostras independentes para a comparação entre os grupos. Para a análise de correlações foi utilizado o teste de correlação de Pearson. Para todas as análises, o nível de significância adotado foi de p < 0,05. Os valores foram expressos em médias e desvios-padrão.

## Resultados

A amostra envolveu 85 pacientes: 55 com DPOCg (média de idade de 62 ± 11,3 anos e de índice de massa corpórea [IMC] de 22,5 ± 3,3 kg/m^2)^ e 30 com DPOCm (média de idade de 66,0 ± 10,1 anos e de IMC de 25,1 ± 2,8 kg/m^2)^. A Tabela 1 apresenta as variáveis analisadas nos dois grupos.

**Tabela 1:** Características gerais da população estudada.

Variável	DPOCm	DPOCg	p
	(n = 30)	(n = 55)	
IMC, kg/m^2^	25,1 ± 2,8	22,5 ± 3,3	0,01*
Idade, anos	66 ± 10	62 ± 11	0,11
SpO_2_ antes do TC6, %	95,0 ± 1,9	95,0 ± 2,1	0,85
SpO_2_ mínima, %	87,3 ± 3,4	85,0 ± 4,3	0,01*
TD4, s	64 ± 36	66 ± 41	0,81
Tmin, s	109 ± 55	168 ± 67	0,01*
TR, s	112,6 ± 28,8	120,2 ± 31,5	0,28
VEF1%	53,1 ± 9,6	28,0 ± 6,2	0,01*
DTC6, m	451 ± 73	435 ± 74	0.35
Inclinação da reta para queda de SpO_2_ ≥ 4%	0,08 ± 0,04	0,08 ± 0,04	0,89
Inclinação da reta para queda máxima da SpO_2_	0,08 ± 0,03	0,07 ± 0,05	0,27
Inclinação da reta para recuperação da SpO_2_	0,07 ± 0,02	0,09 ± 0,04	0,05

**DPOCm:** : DPOC moderada

**DPOCg:** : DPOC grave

IMC : Índice de massa corporal

TD4 : tempo para atingir dessaturação ≥ 4%

Tmin : tempo para dessaturação máxima

TR : tempo de recuperação

DTC6 : distância percorrida no teste de caminhada de seis minutos

Os grupos mostraram-se homogêneos quanto aos parâmetros idade e SpO_2_ antes do TC6. O IMC não estava acima de 30 kg/m² em nenhum dos grupos, valor a partir do qual há influência na espirometria.^(^ ^10^ ^)^ A distância percorrida pelos dois grupos não foi significativamente diferente. Os valores mínimos de SpO_2_ foram significativamente menores no grupo DPOCg (p < 0,014). Houve queda de 4% na SpO_2_ até o primeiro minuto em 63% e em 71% nos grupos DPOCm e DPOCg, respectivamente. O tempo de dessaturação em 4% e o tempo para recuperação não diferiram significativamente entre os grupos; porém, o tempo para atingir o menor valor de SpO_2_ foi maior no grupo DPOCg que no grupo DPOCm (p < 0,001). As inclinações das curvas de SpO_2_ para uma dessaturação de 4%, para a queda máxima e para a recuperação não mostraram diferenças significativas entre os grupos. A variação da SpO_2_ (ΔSpO_2_) entre o inicio do TC6 e a queda máxima foi estatisticamente diferente entre os dois grupos (p = 0,005).

Em relação às correlações (Figura 3), o VEF_1_% apresentou uma correlação moderada positiva com a SpO_2_ mínima (r = 0,356; p < 0,005), uma correlação moderada negativa com a ΔSpO_2_ (r = −0,398; p < 0,001) e uma correlação moderada negativa com o tempo para atingir a SpO_2_ mínima (r = −0,449; p < 0,001).

**Figura 3:** Correlações das variações do VEF1% com a SpO2 mínima (em A), com ΔSpO2 (em B) e com o tempo para atingir SpO2 mínima (Tmin; em C)

A inclinação da queda máxima da SpO_2_ teve uma correlação moderada negativa com o tempo para atingir a SpO_2_ mínima (r = −0,467; p < 0,001), com o tempo para uma dessaturação de 4 % (r = −0,437; p < 0,001) e com o menor valor da SpO_2_ (r = −0,393; p < 0,001). A distância caminhada no TC6 (DTC6) não apresentou correlações significativas com a SpO_2_ e suas variações e nem com o VEF_1_%.

## Discussão

A dessaturação durante o exercício pode ser medida no TC6 e é um índice de valor prognóstico nas patologias intersticiais e na DPOC. Uma queda na SpO_2_ ≥ 4% sugere uma dessaturação importante e se usa para avaliar a necessidade de oxigênio em pacientes com doenças pulmonares crônicas.^(^ ^12^ ^)^ Outro índice de avaliação da capacidade funcional é a DTC6, que tem um valor prognóstico na DPOC.^(^ ^13^ ^)^

Entretanto, a hipoxemia é um dos maiores problemas na medicina respiratória, pois é muito comum em pacientes com doenças pulmonares e a sua rápida avaliação e tratamento são necessários para prevenir danos orgânicos irreversíveis.

A dessaturação ao exercício é comumente observada em pacientes com DPOC; entretanto, os parâmetros clínicos não são capazes de identificar essa alteração. A SpO_2_ < 95% em repouso foi reportada como um preditor para a dessaturação ao exercício, especialmente em pacientes com DLCO comprometida em ≥ 36%.^(^ ^14^ ^)^ Zafar et al.^(^ ^15^ ^)^ não encontraram uma correlação significativa entre a variação (queda) na SpO_2_ e a SpO_2_ em repouso. Em nosso estudo tampouco houve uma correlação significativa entre a SpO_2_ basal e a queda da SpO_2_ (r = 0,08; p = 0,46).

A teoria da intolerância ao exercício na DPOC é de construção multifatorial: o maior trabalho e consumo de oxigênio pela musculatura respiratória, a disfunção da musculatura esquelética dos membros inferiores e o mecanismo de hiperinsuflação pulmonar dinâmica, atuando isoladamente ou em conjunto.^(^ ^8^ ^)^ Zafar et al.,^(^ ^15^ ^)^ utilizando 30 pacientes com DPOC, demonstraram uma boa correlação entre a dessaturação de oxigênio no TC6 e a hiperinsuflação dinâmica, mas sem correlação com a DTC6. Nossos resultados tampouco mostraram uma correlação entre a dessaturação e a DTC6; entretanto, não avaliamos a hiperinsuflação no presente estudo. 

Observamos que a DTC6 não apresentou correlações com as alterações da SpO_2_. Há relatos na literatura mostrando mudanças na musculatura esquelética dos pacientes com DPOC, destacando-se o predomínio das fibras glicolíticas sobre as oxidativas. A partir dessas alterações, os pacientes utilizam predominantemente o metabolismo anaeróbio num precoce nível de esforço,^(^ ^16^ ^,^ ^17^ ^)^ caracterizando uma mudança da rota metabólica e reduzindo a carga aeróbica. A ocorrência de alguns fatores, tais como estresse inflamatório, descondicionamento físico, uso prolongado de corticosteroides e hipoxemia, contribui para a modificação da atividade contrátil do músculo, desencadeando uma série de adaptações que envolvem uma alteração das fibras musculares. Segundo um grupo de autores,^(^ ^18^ ^)^ o trabalho respiratório é duas vezes maior no grupo de pacientes com DPOC que são recrutadores da musculatura abdominal do que no grupo com DPOC não recrutador, associado à maior dispneia e menor tolerância ao exercício. Essa é uma possível explicação para os nossos resultados, pois os pacientes podem possuir uma predominância de fibras glicolíticas e/ou serem do grupo não recrutador da musculatura abdominal.

Estudos prévios^(^ ^19^ ^,^ ^20^ ^)^ mostraram que o tempo de dessaturação durante o TC6 é um indicativo da possibilidade de dessaturação durante as atividades cotidianas, culminando em hipoxemia severa e necessidade de oxigenoterapia. Jenkins e Cecins^(^ ^21^ ^)^ analisaram os eventos adversos durante o TC6 em um grupo de 572 pacientes com DPOC que completaram o TC6; 345 (47%) dos pacientes exibiram dessaturação significativa (queda ≥ 4%). O estudo de Jenkins e Cecins^(^ ^21^ ^)^ destaca a importância do monitoramento contínuo da SpO_2_ no TC6. O sistema de telemetria utilizado no HCPA nos permitiu um seguimento do comportamento da SpO_2_ em tempo real durante o TC6.

Um grupo de autores^(^ ^20^ ^)^ mostrou que, em 83 pacientes com DPOC que realizaram o TC6, 48 apresentaram SpO_2_ < 90% precoce (antes do primeiro minuto) e, em um seguimento de 5 anos, 65% daqueles pacientes migraram para hipoxemia severa com necessidade de oxigenoterapia domiciliar, em contraste com 11% dos pacientes que não apresentaram dessaturação precoce (p < 0,001). A dessaturação precoce também está associada com a dessaturação durante 24 h e durante a maior parte das atividades da vida diária. Na nossa amostra composta de pacientes que apresentaram dessaturação no TC6, notamos que a maioria dos pacientes dos grupos DPOCg e DPOCm apresentou uma dessaturação ≥ 4% no primeiro minuto (71% e 63% dos pacientes, respectivamente), indicando a necessidade de uma avaliação mais rigorosa das atividades de rotina desses indivíduos.

Em um estudo,^(^ ^22^ ^)^ foram estudados 224 pacientes com DPOC divididos em dois grupos: com e sem dessaturação de oxigênio no TC6. Os pacientes foram acompanhados por 3 anos, e foi observado que o VEF_1_ teve uma redução mais rápida no grupo com dessaturação (p = 0,006), sugerindo que a dessaturação ao exercício pode ser um preditor para o declínio da função pulmonar em pacientes com DPOC. Em nosso estudo, o VEF_1_ mostrou-se um bom indicador de dessaturação ao exercício, apresentando uma correlação significativa negativa moderada com a ΔSpO_2_ (r = −0,398; p < 0,001) e com o tempo para atingir a SpO_2_ mínima (r = −0,448; p < 0,001). 

A dessaturação de oxigênio é um parâmetro de monitorização que qualifica o desempenho do paciente no TC6 e contribui para dimensionar o grau de comprometimento da doença no esforço físico. A análise da curva de dessaturação nos permite uma visão completa do tempo que inicia a queda da SpO_2_, da intensidade dessa queda e do tempo necessário para sua recuperação, o que pode nos auxiliar a dimensionar a gravidade do quadro clínico; entretanto, até onde sabemos, nenhum estudo prévio expôs esses dados para comparação.

O presente estudo ressalta a importância da análise da dessaturação de oxigênio com o monitoramento contínuo no TC6 em pacientes com DPOC. No grupo estudado, os pacientes do grupo DPOCg, em comparação aos do grupo DPOCm, atingiram valores menores de SpO_2_, e a maioria apresentou dessaturação precoce (até o primeiro minuto), sugerindo um pior prognóstico. O VEF_1_ mostrou-se um bom marcador para a dessaturação ao exercício, apresentando uma correlação moderada com a SpO_2_ mínima, com a ΔSpO_2_ e com o tempo para atingir a SpO_2_ mínima.
